# Depression As a Consequence of Frontal Lobe Infarction: A Case Report

**DOI:** 10.7759/cureus.6347

**Published:** 2019-12-11

**Authors:** Sidra J Faruqi, Muhammad Y Aziz, Abdur R Memon

**Affiliations:** 1 Neurology, Hamdard University Hospital, Karachi, PAK; 2 Medicine, Hamdard University Hospital, Karachi, PAK

**Keywords:** frontal lobe, depression, behavioural disorders, post stroke depression, stroke, post-stroke depression

## Abstract

Stroke is one of the most important and prevalent causes of morbidity and mortality around the world, with the most common site of vascular lesions being the frontal lobe. Usually, strokes present with motor or speech abnormalities. Depression or other psychiatric disturbances being the sole presenting feature of a stroke is a rare occurrence. Sudden onset of behavioral disturbances should alert the physician to investigate the patient for an underlying cause of his/her disorder. We present the case of a 65-year-old diabetic male who presented with sudden low mood and apathy that caused him severe socio-economic losses. He was initially diagnosed with depressive disorder but failed to respond to treatment. He was investigated, and a computed tomography (CT) scan of the brain led to the correct diagnosis, an old left frontal infarct. In patients with behavioral disorders, which are sudden in onset or not responding to treatment, secondary causes should always be considered.

## Introduction

In developing countries, stroke is the third most important cause of death, hospital admission, and long-term disability. In Pakistan, the crude age- and sex-adjusted stroke incidence is 95 per 100,000 persons per year, with the highest incidence being noted in individuals between 75 and 85 years of age [1]. Stroke usually presents with speech and motor difficulties [2]. However, behavioral abnormalities ranging from mild inappropriate social conduct to outright mania can occur as presenting features of strokes, especially frontal lobe lesions [3]. Often, these occur without any accompanying motor or speech abnormality and are incorrectly labeled and managed as inorganic disorders [4].

## Case presentation

We present the case of a 65-year-old male, a known case of diabetes mellitus, who presented to the outpatient department with generalized weakness, low mood, and lack of interest in daily activities for the last 6 to 8 months. Prior to the onset of his symptoms, he had been working abroad for the last 40 years. He was considered a model employee and was trusted by his superiors in decision-making and financial matters. However, in the 6 months prior to his admission, he had been cheated out of his life savings and had also lost his home. He had been dismissed from his job as well for poor performance and incorrect accounting. At that time, he had been diagnosed as suffering from depressive disorder and had been prescribed anti-depressants, with minimal improvement in symptoms.

On presentation to our hospital, he was vitally stable. His general physical examination yielded no significant findings. In his neurological examination, he was conscious and oriented. His Mini-Mental State Examination score was 25/30 with impaired registration and recall. Examination of his motor, sensory, and cerebellar systems was unremarkable. Mental state examination yielded poor eye contact, low mood, and ideas of worthlessness. No abnormal beliefs or perceptions were elicited.

**Figure 1:**
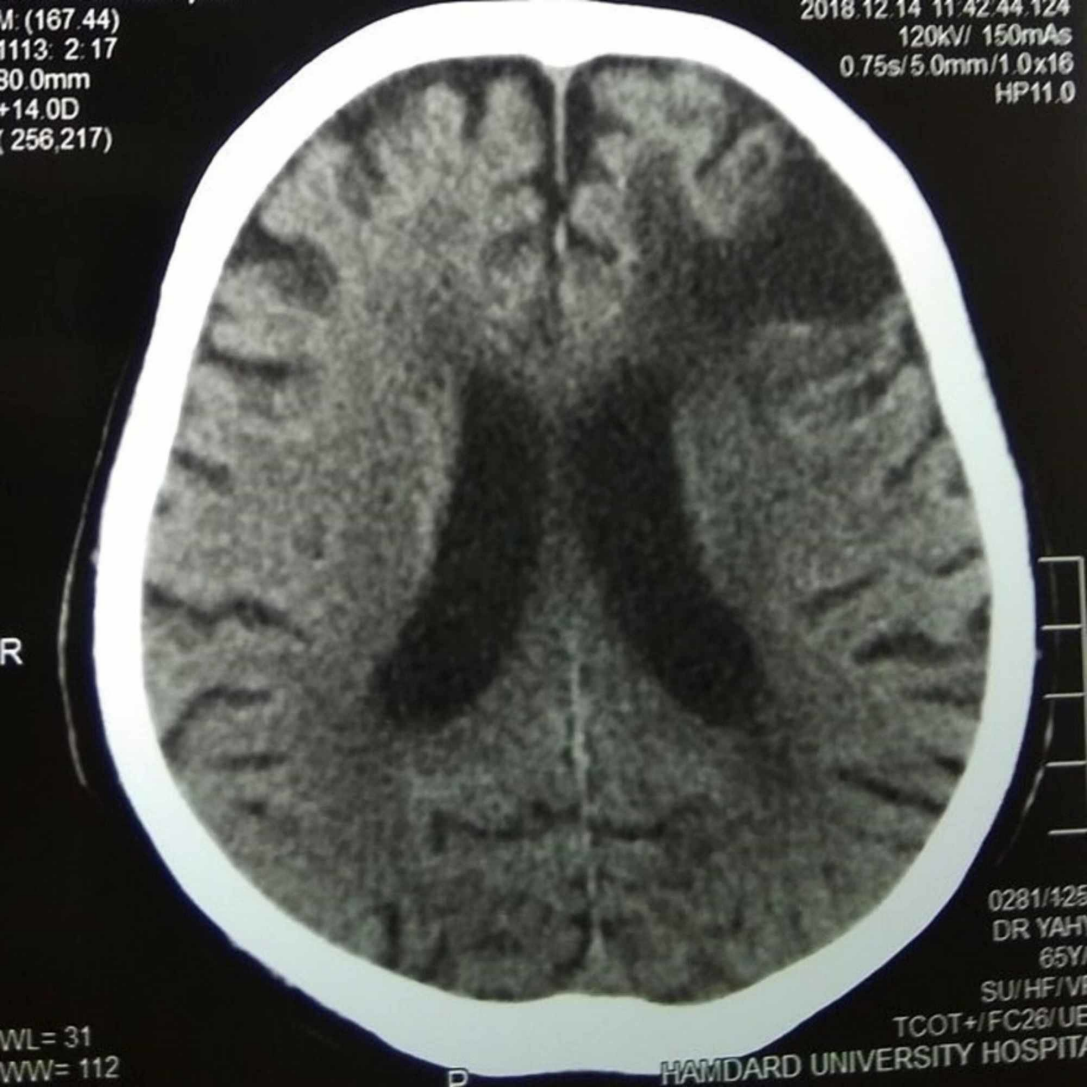
CT scan of the brain (plain, axial view), showing a left frontal infarct

All routine laboratory investigations were normal. His random blood sugar was 140 mg/dL, fasting blood sugar was 98 mg/dL, and glycated hemoglobin (HbA1C) was 6.0%. His fasting lipid profile, serum vitamin B12, serum folate levels, and thyroid function tests were also normal. Screening for viral hepatitis and human immunodeficiency virus (HIV) was negative. A computed tomography (CT) scan of the brain was performed that showed ischemic changes. The most striking finding was an old left frontal infarct with a compensatory dilation of the anterior horn of the left lateral ventricle (Figure 1).

He was treated with aspirin, statins, cognitive behavioral therapy, and sertraline, a selective serotonin re-uptake inhibitor (SSRI). His mood and cognition improved. His recollection and recent memory, which had previously been impaired, also showed mild improvement. He began to take interest in self-care and hygiene and also started to participate in community activities. He was, however, still unable to perform complex calculations like he had done previously as part of his job. 

He has been called for follow-up after 6 months, with a plan to repeat imaging of the brain.

## Discussion

As is evident from this case, imaging of the brain is an important diagnostic modality in patients presenting with psychiatric disturbances, particularly in those with risk factors for stroke, such as diabetes mellitus, or those not responding to treatment.

Post-stroke depression is a common complication of stroke. Studies have shown that 30-60% of stroke patients experience depression, which greatly hampers their rehabilitation [5-6]. Post-stroke depression can occur early after a stroke or several years following a vascular event. A significant increase in mortality has been observed in patients suffering from post-stroke depression [7-8]. However, stroke presenting exclusively as psychiatric disturbances is rare, accounting for only around 3% of all reported cases [9]. The most common psychiatric disturbance reported is depression, although manic disorders, disorders of behavioral regulation, and inappropriate sexual behavior have also been reported as presenting features of acquired cerebral disorders [10-11].

Since the frontal lobe governs memory, emotion, judgment, executive functions, and behavior, a lesion of this lobe is the most common cause of depression or other mood disorders [12]. A lesion of the dominant frontal lobe is more likely to cause these disorders. By virtue of its close connections to the anterior cingulate gyrus and the amygdala, emotional disturbances are also commonly seen in frontal lobe lesions.

A sudden onset of behavioral changes, as in our patient, warrants investigation for a secondary cause of the behavioral disorder. The fact that he did not initially respond to anti-depressants further strengthens this statement.

Our patient was thought to be suffering from depression secondary to the loss of his job and home. However, now it seems that his frontal infarct was, in fact, the cause of both his socio-economic problems and his depression.

## Conclusions

In patients presenting with mood or behavioral disorders, stroke and other structural lesions should always be considered in the differential diagnosis and the patients should undergo laboratory and/or imaging studies if the history or examination point toward an underlying cause of the disorder.
